# The Impact of Adverse Childhood Experiences on Mental Health and Suicidal Behaviors: A Study from Portuguese Language Countries

**DOI:** 10.1007/s40653-023-00540-2

**Published:** 2023-03-31

**Authors:** Daniela Silveira, Henrique Pereira

**Affiliations:** 1 Department of Psychology and Education, University of Beira Interior, Covilhã, Portugal; 2 Research Centre in Sports Sciences, Health Sciences and Human Development (CIDESD), Vila Real, Portugal

**Keywords:** ACEs, Mental health, Suicide ideation, Suicide attempt, Childhood, CPLC

## Abstract

**Background**: Research on adverse childhood experiences (ACEs) demonstrates that they can be associated with physical and mental health problems throughout the lifecourse. However, few studies have examined this topic in the Community of Portuguese Language Countries (CPLC). **Objective**: This study aims to assess the impact of ACEs on mental health and suicidal behaviors in a sample of participants from the CPLC. **Participants and Setting**: The sample consists of 1006 participants aged between 18 and 80 years (mean = 41.76; SD = 14.19). **Methods**: This study used an online survey that included a sociodemographic questionnaire, the Brief Symptom Inventory-18 (BSI-18) to assess somatization, depression, and anxiety symptoms, and overall mental functioning, the Suicidal Behaviors Questionnaire-Revised (SBQ-R) to assess suicidal behaviors, and the Family Adverse Childhood Experiences Questionnaire to assess ACEs. **Results**: Emotional abuse was the most reported ACE (32.7%). Participants from Brazil had higher levels of somatization, depression, anxiety, and suicide ideation and attempt, while participants from Portugal had a higher probability of suicide in the future. ACEs were strong and significant predictors of psychological symptoms and the likelihood of suicide in the future, with emotional abuse and emotional neglect being the domains with the greatest contribution, respectively. **Conclusions**: ACEs are a prevalent and general phenomenon across several countries. It is urgent to alert policymakers and mental health professionals of the need to intervene with children and families to ensure their harmonious and adjusted development, thus promoting quality of life and well-being of populations.

## Introduction

Adverse childhood experiences (ACEs) are frequent and intense experiences of stress during childhood (World Health Organization, 2020), which can have a significant negative impact on the physical and mental health of a person during their lifespan (Felitti et al., 1998). Their implications in health were first described in the study of Felitti and colleagues (1998), where they were viewed as a risk factor (Boullier & Blair, 2018). ACEs can be classified as abuse experiences (emotional, physical, and sexual), dysfunctional family environment (domestic violence, substance abuse by a member of the household, divorce/parental separation, incarceration of a family member, and mental illness/suicide of a family member), and neglect (physical and emotional) (Silva & Maia, 2008).

ACEs have been found to be highly prevalent in community populations and many studies indicate that more than half of the sample reported having experienced at least one domain of childhood adversity (Anda et al., 2006; Campbell et al., 2016; Chang et al., 2019; Chapman et al., 2004; Craig et al., 2020; Felitti et al., 2019; Font & Maguire-Jack, 2016; Merrick et al., 2017; Mersky et al., 2013). Additionally, a high proportion of the population has been exposed to multiple domains of adversity, with the probability of being exposed to more than one domain varying between 65 and 93% (Felitti et al., 2019).

The most frequently reported domains of ACEs, according to the reviewed literature, are emotional abuse (16.2–18.3%) (Riedl et al., 2020; Thompson et al., 2019), substance abuse in the household (25.6%) (Felitti et al., 1998) and parental separation (27.5–43%) (Schilling et al., 2007; Wong et al., 2019), whereas the least reported domains are exposure to criminal behavior in the household (3.4%) (Felitti et al., 1998), death of one of the parents (4.7%) (Thompson et al., 2019), and incarceration of a family member (9%) (Wong et al., 2019).

### Sociodemographic Disparities in ACEs

A substantial body of research has investigated differences in ACEs by demographic variables. Regarding age, for example, the literature suggests that younger ages are associated with the report of a greater number of ACEs (Campbell et al., 2016; Felitti et al., 1998; Riedl et al., 2020), although another study found that older ages report more frequently the domain of physical neglect (Riedl et al., 2020). Being part of an ethnic/racial minority (non-white) (Campbell et al., 2016; Craig et al., 2020; Turner & Butler, 2003) was also associated with the report of a greater number of ACEs. Regarding gender, research has produced mixed results, with some studies reporting higher levels of exposure to ACEs among men (Craig et al., 2020; Mersky et al., 2013; Turner & Butler, 2003), and others report higher levels among women (Campbell et al., 2016; Wong et al., 2019). A consistent finding, however, is that the most prevalent domains of ACEs differ significantly by gender: women report, globally, higher levels of sexual abuse (Edwards et al., 2003; Schilling et al., 2007) and exposure to domestic violence (Edwards et al., 2003), while men report more physical abuse (Chang et al., 2019; Dias et al., 2015; Edwards et al., 2003; Schilling et al., 2007).

Several socioeconomic variables have also been found to be associated with the reporting of a greater number of ACEs, including low socioeconomic status (Turner & Butler, 2003; Wang et al., 2019), low income (Campbell et al., 2016; Font & Maguire-Jack, 2016), living in rural areas (Craig et al., 2020), low educational attainment (Campbell et al., 2016; Felitti et al., 1998; Font & Maguire-Jack, 2016; Wong et al., 2019), and low parental educational attainment (Craig et al., 2020; Turner & Butler, 2003).

### Long-Term Health Impact of ACEs

Multiple studies have sought to identify the main consequences related to the experience of ACEs, and findings indicate that there is a positive correlation between number of ACEs and increased depressive symptoms (Anda et al., 2006; Campbell et al., 2016; Chang et al., 2019; Felitti et al., 1998; Karatekin, 2017; Kong et al., 2021; Merrick et al., 2017; Mersky et al., 2013; Riedl et al., 2020; Schilling et al., 2007; Turner & Butler, 2003; Wong et al., 2019), anxiety (Anda et al., 2006; Karatekin, 2017; Mersky et al., 2013; Riedl et al., 2020), panic reactions (Anda et al., 2006), somatization (Anda et al., 2006; Riedl et al., 2020), stress (Anda et al., 2006; Wong et al., 2019), sleep disturbance (Anda et al., 2006), difficulties in anger control (Anda et al., 2006), and sexual dissatisfaction and promiscuity (Anda et al., 2006; Felitti et al., 2019). There is also a positive correlation between the number of ACEs and suicide attempts (Felitti et al., 1998; Merrick et al., 2017; Thompson et al., 2019), suicidal ideation (Karatekin, 2017; Thompson et al., 2019; Wang et al., 2019; Wong et al., 2019), antisocial behaviors (Schilling et al., 2007), and substance abuse (Anda et al., 2006; Felitti et al., 2019; Merrick et al., 2017; Mersky et al., 2013; Schilling et al., 2007) including alcohol use (Chang et al., 2019) and tobacco use (Felitti et al., 1998). Furthermore, childhood adversity is, in general, related to adopting unhealthy behaviors and, consequently, worse mental (Edwards et al., 2003; Chapman et al., 2004; Merrick et al., 2017; Mersky et al., 2013; Wade et al., 2016) and physical health outcomes, including frequent chronic diseases (Boullier & Blair, 2018; Chang et al., 2019; Kong et al., 2021) and severe obesity (Anda et al., 2006; Felitti et al., 1998). Simultaneously, persons exposed to such adverse events perceive low levels of family support (Jones et al., 2018; Turner & Butler, 2003), self-esteem (Turner & Butler, 2003; Wong et al., 2019), and life satisfaction (Mersky et al., 2013).

Research has tried to understand which domains of ACEs are most associated with certain consequences such as depression, suicidal behaviors, and overall physical and mental health. In relation to depression, emotional abuse is considered by some authors as the biggest risk factor for the disorder (Dias et al., 2015; Chang et al., 2019; Chapman et al., 2004), although other authors recognize a strong relation with other domains, like physical abuse (Campbell et al., 2016; Font & Maguire-Jack, 2016; Pinto et al., 2015), sexual abuse (Campbell et al., 2016; Font & Maguire-Jack, 2016; Giano et al., 2021), mental illness of a family member (Campbell et al., 2016; Font & Maguire-Jack, 2016; Giano et al., 2021; Pinto et al., 2015), substance abuse (Campbell et al., 2016; Font & Maguire-Jack, 2016; Pinto et al., 2015) and incarceration of a household member (Campbell et al., 2016). On the other hand, in a study done by Merrick and colleagues (2017), incarceration of a household member was the only domain not significantly associated with depressive symptoms. Concerning suicide attempt, emotional abuse is also considered to be the main risk factor, (Merrick et al., 2017; Thompson et al., 2019), although sexual and physical abuse, suicide attempt of a family member (Thompson et al., 2019), physical neglect, substance abuse, and family history of mental illness are also well-documented risk factors (Pinto et al., 2015). The ACEs risk factors for suicidal ideation mimic those of suicide attempt, but also include emotional neglect, isolation/rejection by peers (Wang et al., 2019), and incarceration of a family member (Thompson et al., 2019). Finally, in the realms of general mental and physical health, Riedl and colleagues (2020) concluded that emotional abuse is also the domain most consistently associated.

The literature is solid in pointing that ACEs can cause profound, lasting, and cumulative damage (Boullier & Blair, 2018; Chapman et al., 2004; Felitti et al., 1998) to child and adolescent health, predisposing them to various challenges in later stages of development (Chang et al., 2019). However, the way ACEs lead to such implications is not established, as there are several mediating factors. One explanation is that early adversity and the high stress levels to which these children and adolescents are exposed disturb the normal development of identity tasks (Turner & Butler, 2003) and interpersonal relationship skills (Basto-Pereira & Maia, 2019; Turner & Butler, 2003). This, on the one hand, interferes with their ability to establish lasting bonds and, on the other, promotes dysfunctional and antisocial behaviors (Anda et al., 2006; Basto-Pereira & Maia, 2019). In this sense, they may feel less capable and with fewer resources, developing negative attributions about themselves (Turner & Butler, 2003) and reacting more intensely and negatively to daily stressful situations (Kong et al., 2021). To deal with stress, they may adopt risk behaviors (e.g., smoking, drinking alcohol or antisocial behavior) due to their immediate beneficial effects, which harm health and tend to become chronic (Boullier & Blair, 2018; Felitti et al., 2019). These harmful risk behaviors, in addition to the decreased perceived social support that people with ACEs tend to report (Jones et al., 2018), lead to greater adversity and poorer health in adulthood (Jones et al., 2018; Boullier & Blair, 2018).

### Community of Portuguese Language Countries (CPLC)

The Community of Portuguese Language Countries (CPLC) has a population of close to 250 million and occupies an area of about 10.7 million square kilometers across four continents (CPLP, 2022). Despite being geographically separated, this community of countries converges in the same language – the Portuguese language. This unifying point has justified the growing commitment of member countries to joint action in the most diverse areas, namely education and health (CPLP, 2022). However, despite the shared language, countries within the CPLC have differing socioeconomic development patterns, which may influence the occurrence of ACEs.

African Countries with Portuguese as an Official Language (PALOP) are part of the CPLC, however, form a distinct group with its characteristics. This group of African countries, including Angola, Mozambique, Guinea-Bissau, Cape Verde, and São Tomé and Príncipe, share a similar historical trajectory, marked by civil wars and poverty, due to the colonization experience and the consequent independence from Portugal (Liberato, 2021). Thus, although the expected uniqueness of each country, it is also likely that this common historical context impacts the occurrence of ACEs.

In this way, despite the cultural diversity existing in the CPLC, their inclusion in this study seems relevant to us to be able to explore, in greater detail, possible points of approximation or separation across countries. In the case of the PALOP, their inclusion is reinforced by the lack of literature on ACEs undertaken in their population, to the best of our knowledge.

In Portugal, according to a report from the Portuguese Association for Victim Support (2021), 1841 children were reported to be victims of some type of abuse in 2020. These child victims were predominantly female, with an average age of 10 years, and were commonly the child of the perpetrator. Another report from the National Commission for the Promotion of the Rights and Protection of Children and Youth (2021) found that, in the same year, 11,995 cases of neglect, 13,363 cases of domestic violence, 1,711 cases of physical abuse, 1,192 cases of emotional abuse, and 712 cases of sexual abuse were reported in Portugal. Research in this area in Portugal has concluded that ACEs are frequent and predict depressive symptoms and suicide attempts (Pinto et al., 2015), and are related to psychosocial problems and persistent criminal behavior in young adulthood (Basto-Pereira et al., 2016; Basto-Pereira & Maia, 2019). Additionally, results have indicated that maltreatment in childhood and adolescence have lasting effects which result in the adoption of antisocial behaviors (Braga et al., 2018).

In Brazil, research regarding ACEs is mainly focused on young ages and less is known about the adult population. However, a recent study concluded that the prevalence of smoking habits and respective dependence in adulthood are significantly associated with exposure to ACEs (Maia-Silva et al., 2021).

Therefore, considering the aforementioned circumstances of the CPLC and the impact of ACEs on individual, group, and social levels of mental health, this study aims to assess the impact of ACEs on mental health and suicidal behaviors in a sample of participants from the CPLC. More specifically it aims to:

- Describe the sociodemographic characteristics of the sample;
- Compare the prevalence of ACEs, mental health levels, and suicidal behaviors by country of residence;
- Identify which domains of ACEs are the most prevalent in our sample;
- Measure the correlations between ACEs and age, mental health, and suicidal behaviors;
- Assess which domains of ACEs are most predictive of mental health and suicidal behaviors in the present;
- Assess the predictive power of the domains of ACEs in explaining mental health and suicidal behaviors of the sample.

## Methods

### Participants

The sample consists of 1006 participants aged between 18 and 80 years (Mean = 41.76; SD = 14.19), of which 576 identified as female, 424 as male, and 6 as another gender. Most participants lived in Brazil (40.7%) and about half were white/European. Regarding sexual orientation, 87.5% self-identified as heterosexual, 6.5% as bisexual, and 6% as gay or lesbian. The majority of the sample was professionally active (60.4%), lived in a large urban area (56%), was of average socioeconomic status (58%), and had obtained at least a bachelor’s degree (90%). In terms of marital status, 37% of participants were married to a person of the opposite sex and 32.3% lived with their spouse. Finally, 67.2% reported being affiliated with a religion. Table 1 shows the sociodemographic characteristics of the sample in greater detail.

**Table 1:** Sociodemographic characteristics (n = 1006; M_age_=41.76; SD_age_=14.19)

		n	%	
Gender	Male Female Other	424 576 6	42.1 57.3 0.6	
Sexual orientation	Heterosexual Bisexual Gay/Lesbian	880 66 60	87.5 6.5 6.0	
Country of residence	Portugal Brazil PALOP	296 409 301	29.4 40.7 29.9	
Race/ethnicity	White/European African/Black Mixed Race	502 253 251	50.0 25.1 24.9	
Professional status	Employed Unemployed Student Student-worker Self-employed Retired Other	608 47 143 82 65 52 9	60.4 4.7 14.2 8.1 6.4 5.2 0.9	
Education	High school or less Bachelor’s degree Master’s degree Doctorate/Ph.D.	100 249 339 318	10.0 24.7 33.7 31.6	
Socioeconomic status	Low Lower-middle Middle Upper-middle High	35 109 584 227 51	3.5 10.9 58.0 22.5 5.1	
Place of residence	Small rural area Large rural area Small urban area Large urban area	71 27 345 563	7.0 2.7 34.3 56.0	
Marital status	Single Dating Same-sex marriage Opposite-sex marriage De facto same-sex union De facto opposite-sex union Separated/divorced from same-sex person Separated/divorced from opposite-sex person Widower of opposite-sex person	221 169 14 372 12 134 11 60 13	21.9 16.8 1.4 37.0 1.2 13.3 1.1 5.9 1.3	
Living situation	Living alone Living with a partner Living with spouse Living with children Living with parents/father/mother Living with friends Other	172 146 325 73 169 47 74	17.1 14.5 32.3 7.2 16.8 4.7 7.3	
Religion	Yes No	676 330	67.2 32.8	

### Measurement Instruments

A sociodemographic questionnaire was used to collect age, gender, sexual orientation, country of residence, race/ethnicity, professional status, educational attainment, socioeconomic status, place of residence, marital status, living situation, and religion.

To assess the mental health of the sample, the Brief Symptom Inventory-18 (BSI-18) (Nazaré et al., 2017), an instrument for screening psychological distress, was used. Its 18 items are evaluated on a Likert scale ranging from *(0) Never* to *(4) Always*, with the participants’ responses referring to their experiences of psychopathological symptoms in the last week. The BSI-18 has three subscales, corresponding to three domains of psychopathological symptoms: somatization, depression, and anxiety. The scale also can be used to calculate a Global Severity Index (GSI) by summing the scores of the 18 items, which reflects the global level of psychological distress experienced by the participants. The somatization subscale assesses the malaise resulting from manifestations of autonomously regulated systems (e.g., cardiovascular, gastrointestinal, etc.); the depression subscale assesses symptoms indicative of depressive disorders (e.g., lack of motivation and interest in activities, loss of energy, suicidal ideation, etc.); and the anxiety subscale assesses symptoms associated with generalized anxiety or panic attacks (e.g., nervousness, tension, motor restlessness, apprehension, etc.). Higher scores on the three subscales and the GSI reflect more intense psychopathological symptoms. Cronbach’s alphas for the total scale and the three subscales were calculated to assess the instrument’s internal consistency, obtaining results of 0.93, 0.78, 0.86, and 0.87 for the total scale and the somatization, depression, and anxiety subscales, respectively. Thus, the instrument showed good internal consistency.

The original version of the Suicidal Behaviors Questionnaire-Revised (SBQ-R) (Osman et al., 2001), validated in its Portuguese-language version (Campos & Holden, 2019), was used to assess suicidal behavior. The five items of the SBQ-R assess suicide ideation, attempt, and intention and are each evaluated on a Likert scale. The total score for the scale ranges from 3 to 18 points, and the cut-off point for screening for the non-clinical Portuguese population is 7.

Finally, the Portuguese-language version of the Family Adverse Childhood Experiences Questionnaire (Silva & Maia, 2008) assessed the report of ACEs through 10 items, adapted from the original 77. Responses were evaluated on a Likert scale from *(0) Never* to *(9) Many times* on an increasing frequency scale. The 10 items constitute 10 domains of adversity: emotional abuse, physical abuse, sexual abuse, emotional neglect, physical neglect, divorce or parental separation, exposure to domestic violence, substance abuse in the family environment, mental illness or suicide of a family member, and incarceration of a family member. The score is obtained by measuring the means of participants’ responses, with higher scores describing a higher frequency of exposure to ACEs. To simplify the scoring, we transformed this variable into four categories as follows: no exposure (0), mild exposure (1–3), moderate exposure (4–6), and severe exposure (7–9).

### Procedures

The present study was approved by the Ethics Committee of the University of Beira Interior (CE-UBI-Pj-2021-047), ensuring the ethical principles of informed consent, anonymity, confidentiality, beneficence and respect for the integrity of participants, and right to withdraw from the study.

For the purpose of the investigation, a website was designed to disseminate the online survey through mailing lists and social networks from May to October 2021. With the collected information, a database was built in IBM SPSS Statistics (version 28, Armonk, NY), where the data were cleaned and analyzed according to research objectives. Both databases were encrypted, making it impossible to access any identifying participant information, such as IP addresses.

### Data Analysis

The collected data were submitted to multiple statistical analyses, according to the objectives defined for this investigation. Descriptive statistics (mean, standard deviation, frequencies, and percentages) were conducted to describe the prevalence of ACEs, levels of psychological symptoms, and reporting of suicidal behavior. A Pearson chi-square test was used to compare differences in ACEs and were conducted one-way ANOVAs to compare differences in means of somatization, depression, and anxiety and suicidal behaviors by countries of residence. To assess the strength and direction of possible associations between age, total BSI-18 score, probability of future suicide, and the ten domains of childhood adversity, Pearson’s correlations were calculated. Finally, two multiple linear regressions were performed to assess the predictive power of ACEs on mental health and participants’ probability of suicide. For all analyses, a p-value of < 0.05 was considered statistically significant.

## Results

Table 2 presents the prevalence of ACEs in the overall sample and by country of residence. Emotional abuse was the most reported domain, with 32.7% of all participants reporting having experienced it at least once. Mental illness or suicide of a family member was the second most reported domain (30.8%), followed by emotional neglect (29.9%). Other ACEs and their respective prevalence can be seen in more detail in Table 2.

**Table 2:** Prevalence of ACEs by country of residence

		Absence	Mild	Moderate	Severe	χ^2^(df)	*p*
Emotional abuse	Portugal Brazil PALOP Total	215 (21.4%) 247 (24.5%) 215 (21.4%) 677 (67.3%)	43 (4.3%) 82 (8.1%) 56 (5.6%) 181 (18.0%)	18 (1.8%) 38 (3.8%) 11 (1.1%) 67 (6.7%)	19 (1.9%) 44 (4.4%) 17 (1.7%) 81 (8.0%)	21.980(6)	0.001*
Physical abuse	Portugal Brazil PALOP Total	243 (24.1%) 308 (30.6%) 220 (21.9%) 772 (76.7%)	26 (2.6%) 55 (5.5%) 57 (5.7%) 139 (13.8%)	15 (1.5%) 21 (2.1%) 8 (0.8%) 45 (4.5%)	10 (1.0%) 27 (2.7%) 13 (1.3%) 50 (5.1%)	19.213(6)	0.004*
Sexual abuse	Portugal Brazil PALOP Total	256 (25.4%) 300 (29.8%) 233 (23.2%) 789 (78.4%)	23 (2.3%) 57 (5.8%) 41 (4.2%) 123 (12.3%)	7 (0.7%) 26 (2.6%) 10 (1.0%) 44 (4.4%)	10 (1.0%) 25 (2.5%) 14 (1.4%) 50 (5.0%)	20.015(6)	0.003*
Emotional neglect	Portugal Brazil PALOP Total	208 (20.7%) 271 (26.9%) 226 (22.5%) 705 (70.1%)	43 (4.3%) 65 (6.5%) 39 (3.9%) 147 (14.6%)	28 (2.8%) 43 (4.3%) 16 (1.6%) 88 (8.7%)	16 (1.6%) 33 (3.3%) 16 (1.6%) 66 (6.6%)	11.256(6)	0.081
Physical neglect	Portugal Brazil PALOP Total	284 (28.2%) 360 (35.8%) 256 (25.4%) 900 (89.4%)	8 (0.8%) 35 (3.5%) 28 (2.8%) 72 (7.2%)	2 (0.2%) 9 (0.9%) 9 (0.9%) 20 (2.0%)	2 (0.2%) 6 (0.6%) 6 (0.6%) 14 (1.4%)	19.529(6)	0.003*
Divorce/parental separation	Portugal Brazil PALOP Total	253 (25.1%) 287 (28.5%) 209 (20.8%) 749 (74.4%)	27 (2.7%) 67 (6.7%) 58 (5.8%) 153 (15.2%)	7 (0.7%) 25 (2.5%) 19 (1.9%) 52 (5.2%)	7 (0.7%) 31 (3.1%) 13 (1.3%) 52 (5.2%)	32.610(6)	.ooo**
Domestic violence in the household	Portugal Brazil PALOP Total	251 (25.5%) 344 (34.9%) 252 (25.6%) 866 (86.0%)	23 (2.3%) 34 (3.4%) 24 (2.4%) 82 (8.1%)	10 (1.0%) 8 (0.8%) 8 (0.8%) 26 (2.6%)	7 (0.7%) 19 (1.9%) 6 (0.6%) 32 (3.2%)	5.980(6)	0.425
Substance abuse in the household	Portugal Brazil PALOP Total	236 (23.5%) 285 (28.3%) 220 (21.9%) 741 (73.7%)	21 (2.1%) 54 (5.4%) 44 (4.4%) 120 (11.9%)	18 (1.8%) 32 (3.2%) 19 (1.9%) 70 (7.0%)	18 (1.8%) 41 (4.1%) 16 (1.6%) 75 (7.5%)	16.709(6)	0.010*
Mental illness or suicide of a family member	Portugal Brazil PALOP Total	192 (19.1%) 263 (26.1%) 241 (24.0%) 696 (69.2%)	50 (5.0%) 77 (7.6%) 31 (3.1%) 158 (15.7%)	34 (3.4%) 39 (3.9%) 17 (1.7%) 91 (9.0%)	17 (1.7%) 34 (3.4%) 10 (1.0%) 61 (6.1%)	27.775(6)	0.000**
Incarceration of a family member	Portugal Brazil PALOP Total	275 (27.8%) 319 (32.3%) 215 (21.7%) 824 (81.8%)	13 (1.3%) 56 (5.7%) 56 (5.7%) 127 (12.6%)	1 (0.1%) 16 (1.6%) 14 (1.4%) 31 (3.1%)	1 (0.1%) 13 (1.3%) 10 (1.0%) 24 (2.4%)	52.291(6)	0.000**

* p < .05

**p < .001

The average levels of psychopathological symptoms in the sample overall and by country of residence are described in Table 3. Regarding the Portuguese sample, the means found agree with the expected means for community samples (Nazaré et al., 2017). The results obtained for the comparison of means between the three groups showed statistically significant differences for the means of somatization levels (F(2.988) = 4.543; p < .05), depression (F(2;977) = 9,716; p < .001), anxiety (F(2;974) = 17.688; p < .001), and total BSI-18 score (F(2;991) = 11.101; p < .001). Residents of Brazil had higher average levels of somatization (M = 0.64; SD = 0.69), depression (M = 1.04; SD = 0.82), anxiety (M = 1.12; SD = 0.86), and total BSI-18 score (M = 0.93; SD = 0.71) than their counterparts from Portugal and PALOP.

**Table 3:** Results for symptoms of somatization, depression, and anxiety by country of residence

		Mean (SD)	F(df)	*p*
Somatization	Portugal Brazil PALOP Total	0.51 (0.55) 0.64 (0.69) 0.52 (0.57) 0.56 (0.62)	4.543 (2;988)	0.011*
Depression	Portugal Brazil PALOP Total	0.84 (0.75) 1.04 (0.82) 0.79 (0.73) 0.91 (0.78)	9.716 (2;977)	0.000**
Anxiety	Portugal Brazil PALOP Total	0.92 (0.70) 1.12 (0.86) 0.77 (0.69) 0.96 (0.78)	17.688 (2;974)	0.000**
Total	Portugal Brazil PALOP Total	0.76 (0.58) 0.93 (0.71) 0.71 (0.63) 0.81 (0.66)	11.101 (2;991)	0.000**

* p < .05

**p < .001

Table 4 describes the average scores for the five items that assess suicidal behavior in the sample overall and by country of residence. Statistically significant differences were found in lifetime suicidal ideation (F(2;945) = 11.441; p < .001) and in suicidal ideation in the last year (F(2;987) = 7.112; p < .001) by country of residence, with Brazilian residents showing higher averages for both (lifetime: M = 1.56, SD = 0.66; last year: M = 1.44; SD = 0.92). However, Portuguese residents had significantly higher average scores for probability of suicide in the future (M = 1.44; SD = 0.97) (F(2.981) = 5.525; p < .05).

**Table 4:** Results for suicidal behaviors by country of residence

		Mean (SD)	F (df)	*p*
Lifetime suicide ideation	Portugal Brazil PALOP Total	1.48 (0.60) 1.56 (0.66) 1.33 (0.55) 1.46 (0.61)	11.441 (2;945)	0.000**
Lifetime suicide attempt	Portugal Brazil PALOP Total	1.17 (0.58) 1.20 (0.62) 1.16 (0.55) 1.18 (0.59)	0.451 (2;989)	0.637
Suicide ideation in the past year	Portugal Brazil PALOP Total	1.33 (0.86) 1.44 (0.92) 1.20 (0.63) 1.33 (0.83)	7.112 (2;987)	0.000**
Suicide attempt in the past year	Portugal Brazil PALOP Total	1.06 (0.38) 1.03 (0.23) 1.08 (0.42) 1.05 (0.34)	1.913 (2;986)	0.148
Probability of suicide in the future	Portugal Brazil PALOP Total	1.44 (0.97) 1.38 (0.99) 1.20 (0.71) 1.35 (0.91)	5.525 (2;981)	0.004*

* p < .05

**p < .001

Table 5 presents the correlation matrix between the following variables: age, total BSI-18 score, probability of future suicide, and scores obtained for each ACE domain. The results showed that the probability of suicide in the future was significantly and positively correlated with total BSI-18 score (r = .357; p < .001). All ACEs were significantly correlated with each other (p < .001), with emotional and physical abuse having the strongest correlation (r = .678). There was also a strong correlation between emotional neglect and emotional (r = .494), physical (r = .413), and sexual (r = .312) abuse and a strong correlation between substance abuse in the household and exposure to domestic violence (r = .428). All correlations can be seen in greater detail in Table 5.

**Table 5:** Results for correlation values among variables

	1	2	3	4	5	6	7	8	9	10	11	12	13
1 – Age	–												
2 – BSI-18 total score	− 0.238**	–											
3 - Probability of suicide in the future	− 0.073*	0.357**	–										
4 - Emotional abuse	− 0.082*	0.248**	0.174**	–									
5 - Physical abuse	0.028	0.190**	0.116**	0.678**	–								
6 - Sexual abuse	− 0.041	0.169**	0.174**	0.264**	0.250**	–							
7 - Emotional neglect	− 0.109**	0.269**	0.229**	0.494**	0.413**	0.312**	–						
8 - Physical neglect	− 0.030	0.135**	0.149**	0.254**	0.322**	0.294**	0.318**	–					
9 – Divorce / parental separation	− 0.111**	0.152**	0.102**	0.238**	0.233**	0.197**	0.274**	0.273**	–				
10 - Domestic violence in the household	− 0.047	0.099**	0.074*	0.326**	0.312**	0.259**	0.291**	0.294**	0.322**	–			
11 - Substance abuse in the household	− 0.072*	0.129**	0.086**	0.270**	0.229**	0.214**	0.237**	0.315**	0.254**	0.428**	–		
12 - Mental illness or suicide of a family member	− 0.062	0.151**	0.172**	0.237**	0.213**	0.187**	0.209**	0.233**	0.199**	0.266**	0.354**	–	
13 - Incarceration of a family member	− 0.034	0.149**	0.063*	0.191**	0.198**	0.215**	0.176**	0.296**	0.228**	0.307**	0.261**	0.173**	–

* p < .05

**p < .001

To assess the contribution of sociodemographic variables and ACEs on psychopathological symptoms, a hierarchical multiple linear regression was performed (see Table 6). The first model, which included only sociodemographic variables (age, gender, sexual orientation, country of residence, race/ethnicity, socioeconomic status, and marital status), explained 11.8% of the variance in participants’ psychopathological symptom scores. In the second model, where we added the 10 ACE domains, the variance explained by the model increased to 21.0%, which demonstrates the impact of these experiences on the mental health of participants. Of all adversity domains, emotional abuse (β = 0.125; p < .05), emotional neglect (β = 0.124; p < .05), and incarceration of a family member (β = 0.120; p < .001) were the strongest predictors. Other predictors can be observed in more detail in Table 6.

**Table 6:** Results for the hierarchical multiple linear regression predicting anxiety, depression, and somatization symptoms

	Model 1			Model 2		
Variable	B	SE B	β	B	SE B	β
Age	− 0.007	0.002	− 0.160**	− 0.007	0.002	− 0.154**
Gender	0.182	0.044	0.141**	0.153	0.043	0.119**
Sexual orientation	0.104	0.042	0.084*	0.014	0.042	0.011
Country of residence	0.014	0.032	0.016	0.007	0.031	0.008
Race/ethnicity	− 0.012	0.028	− 0.015	− 0.042	0.027	− 0.054
Socioeconomic status	− 0.167	0.028	− 0.204**	− 0.142	0.027	− 0.173**
Marital status	0.004	0.011	0.015	0.007	0.011	0.022
Emotional abuse				0.033	0.012	0.125*
Physical abuse				0.006	0.014	0.021
Sexual abuse				0.015	0.011	0.049
Emotional neglect				0.034	0.011	0.124*
Physical neglect				0.007	0.020	0.014
Divorce/parental separation				0.018	0.011	0.058
Domestic violence in the household				− 0.045	0.015	− 0.116*
Substance abuse in the household				0.000	0.010	0.002
Mental illness or suicide of a family member				0.012	0.010	0.043
Incarceration of a family member				0.052	0.015	0.120**
R^2^	0.118			0.210		
F for change in R^2^	16.298**			13.219**		

* p < .05

**p < .001

Another hierarchical multiple linear regression was performed to assess the contribution of the same variables on the probability of suicide in the future (see Table 7). The first model with sociodemographic variables explained 5.1% of the variance in probability of suicide. After the inclusion of ACEs, the model was able to explain 12.5% of the variance in probability of suicide in the future. The fact that the variability explained by the model more than doubled when ACEs were added demonstrates the relevance of these experiences in the area of suicide, especially regarding suicide risk in the future. Emotional neglect (β = 0.148; p < .001), sexual abuse (β = 0.115; p < .05), and mental illness/suicide of a family member (β = 0.092; p < .05) proved to be the strongest predictors among the 10 domains.

**Table 7:** Results for the multiple linear regression predicting suicidal behaviors

	Model 1			Model 2		
Variable	B	SE B	β	B	SE B	β
Age	− 0.001	0.002	− 0.018	0.000	0.002	− 0.003
Gender	0.012	0.064	0.007	− 0.034	0.063	− 0.019
Sexual orientation	0.305	0.061	0.178**	0.189	0.061	0.110*
Country of residence	− 0.088	0.046	− 0.074	− 0.090	0.046	− 0.076*
Race/ethnicity	0.001	0.041	0.001	− 0.017	0.040	− 0.015
Socioeconomic status	− 0.114	0.040	− 0.100*	− 0.085	0.040	− 0.074*
Marital status	0.011	0.016	0.026	0.009	0.016	0.020
Emotional abuse				0.028	0.018	0.076
Physical abuse				− 0.041	0.021	− 0.093
Sexual abuse				0.051	0.016	0.115*
Emotional neglect				0.058	0.016	0.148**
Physical neglect				0.060	0.029	0.079*
Divorce/parental separation				0.020	0.016	0.044
Domestic violence in the household				− 0.029	0.022	− 0.054
Substance abuse in the household				− 0.025	0.015	− 0.066
Mental illness or suicide of a family member				0.036	0.014	0.092*
Incarceration of a family member				0.012	0.022	0.020
R^2^	0.051			0.125		
F for change in R^2^	6.556**			7.059**		

* p < .05

**p < .001

## Discussion

The main purpose of the present study was to assess the impact of ACEs on mental health and suicidal behavior in a sample of participants from the CPLC. The results show that the prevalence of ACEs in the overall sample is relatively high, ranging from 10.6% (physical neglect) to 32.7% (emotional abuse), with emotional abuse being the most reported. These results are similar to those found in other studies (Riedl et al., 2020; Thompson et al., 2019), demonstrating that ACEs are a worrying phenomenon that impact a wide range of cultural populations.

In terms of psychological symptoms, we concluded that although the overall sample does not constitute a clinical sample and psychological symptoms were self-reported, participants from Brazil demonstrated significantly higher levels of somatization, depression, and anxiety symptoms. One potential explanation for this disparity is the country’s socioeconomic and sociopolitical context, where high rates of violence and marked social and racial inequalities (Madeira & Gomes, 2018; Weichert, 2017), may contribute to the intensification of psychological symptoms. This explanation would support the fact that depression and other common mental illnesses are more prevalent in Brazil than in Portugal, for instance (WHO, 2017).

Levels of suicidal ideation throughout the lifespan and in the previous year were also significantly higher in participants from Brazil, which is consistent with the fact that Brazil represents one of the ten countries in the world with the most recorded cases of suicide (Barbosa & Teixeira, 2021). In contrast, the probability of suicide in the future was significantly higher among participants from Portugal, which supports the previous research indicating that higher-income countries have higher suicide rates than low- or middle-income countries (Klonsky et al., 2016; WHO, 2014). One reason for this is that individuals in higher-income countries may be exposed to higher pressure from social and/or economic competition. This pressure could increase the likelihood of suicidal behavior in the future, as suicide may seem to be a way to minimize the suffering of current stressors. On the other hand, suicide ideation and attempt, and the respective evolution from ideation to attempt, must be understood as distinct processes with distinct predictors and explanations (Klonsky et al., 2016). Therefore, high levels of ideation may not always lead to high levels of attempt. The fact that Portuguese residents had lower rates of suicidal ideation and attempt, but higher probability of suicide in the future than Brazilian and PALOP residents can also be explained by the underreporting or biased classification of deaths by suicide in Portugal. Because suicide is a sensitive and little discussed topic in Portugal, deaths by suicide are often registered as deaths by “undetermined causes” or “other causes”, which makes this phenomenon, in some way, masked and/or invisible in the country (Nunes, 2018; National Program for Mental Health, 2015; WHO, 2014). This interferes with the way people recognize, think, and communicate suicide ideation and attempt because the report of those experiences implies some level of insight, while probability of suicide represents a form of projection into the future, which may lead to the underreporting of such experiences in surveys.

The manifestation of suicidal behavior also differs according to sociodemographic variables. For example, suicide in Portugal is more frequent among males, older age groups, and in certain geographical areas with cultural specificities (Nunes, 2018), demonstrating how social variables seem to impact suicidal behavior.

A significant, positive, and strong correlation was found between psychological symptoms and the likelihood of suicide in the future, confirming the role of mental health in the manifestation of suicidal behavior, as a multifactorial phenomenon (Brådvik, 2018). These results also demonstrated a strong association between physical and emotional abuse, suggesting that the experience of multiple types of adversity in childhood is highly associated (Descartes et al., 2020; Edwards et al., 2003; Felitti et al., 2019; Karatekin, 2017; Mersky et al., 2013; Riedl et al., 2020), and, specifically, that physical abuse co-occurs with psychological abuse (Descartes et al., 2020; Dias et al., 2015). Exposure to domestic violence was also strongly associated with substance abuse in the household, an association also found in previous studies (Canfield et al., 2020; Fals-Stewart, 2003). This result may be explained by the fact that substance abuse (e.g., alcohol) may affect brain structures responsible for the inhibitory control of behaviors, inducing primitive forms of behavior, such as aggressive impulses. Also, they affect cognitive functioning and processing, making individuals less capable to process information, which can lead them to act aggressively (Chermack & Giancola, 1997).

ACEs proved to be strong predictors of both psychological symptoms and suicidal behavior in our sample. Emotional abuse was the strongest predictor in explaining psychological symptoms, similar to the results found in other studies (Dias et al., 2015; Chang et al., 2019; Chapman et al., 2004), which considered it as the main risk factor for the development of depression. This result highlights the importance of emotional issues and particularly emotional abuse in children’s mental health (Dias et al., 2015), even though they are often overlooked compared to physical abuse. Regarding suicidal behavior, the strongest predictor in our sample was emotional neglect. This differs from results of other studies, which have found that emotional (Merrick et al., 2017; Thompson et al., 2019), sexual, and physical abuse, attempted suicide by a family member (Thompson et al., 2019), physical neglect, substance abuse in the household, and family history of mental illness (Pinto et al., 2015) are the strongest predictors of suicidal behavior. Although this is not one of the predictors usually mentioned in the literature, we believe that characteristics of emotional neglect, including passivity, indifference, and non-responsiveness to needs, can trigger feelings of hopelessness and lack of interest in life. Also, since suicidal behavior is a multifactorial phenomenon, it’s expected that the predictors for its occurrence may vary by population and culture as well. Thus, this study reinforces the need to consider the emotional variables of childhood in order to promote a more harmonious child development, which is demonstrably a predictor of less mental health challenges in adulthood (Dias et al., 2015).

Despite its contributions, our study also had some limitations. The sample was collected online and is highly differentiated, so it is neither representative nor generalizable to the populations studied. Data collection through a questionnaire may be subject to the effects of social desirability and, since adverse experiences were retrospectively reported, the respondents may be subject to memory lapses and/or biases. Additionally, although we value the importance of the contribution of the different CPLC included in the study, their inclusion may be a limitation, as the regions face quite different demographic and sociopolitical realities, especially in terms of human development indexes, thus limiting the drawing of conclusions. Furthermore, the cross-sectional nature of this study makes it impossible to monitor changes over time and establish causal relationships between ACEs and the mental health and suicidal behavior of the sample. Finally, the lack of prior research regarding PALOP makes it difficult to compare our results with other literature.

Although our study had limitations, it also has many strengths, especially regarding the consequences for the practices of promotion, prevention, and intervention in mental health. The results emphasize the urgency to create and implement health, educational, childhood, and family policies to protect children and adolescents in various countries. They also emphasize the need for government accountability, as governments are active agents in the pursuit of children’s dignity and rights. In terms of preventing mental health problems and suicide, it is important to develop mechanisms for early identification, assessment, and intervention, focused on preventing child abuse and minimizing potential harmful consequences. This study also provides knowledge of which important areas of psychological functioning are influenced by ACEs, which may help mental health professionals to develop assessment and intervention programs more adjusted to the needs of populations in each geographic area.

Future research should include qualitative studies that assess other developmental indicators, thus promoting broader and more global knowledge on this topic. Studies that work directly with children and adolescents may also be relevant so that the report of ACEs is relative to the present and not retrospective. Longitudinal studies, which allow for the monitoring of changes throughout development and the establishment of causal relationships, will also be important in future research. Finally, future research should try to include more representative samples in order to draw generalizations for the population.

## Conclusion

It is concluded that ACEs are a prevalent and general phenomenon across several countries, with a significant negative impact on mental health and suicidal behavior. Therefore, it is urgent to alert policy makers and mental health professionals of the need to intervene with children, young people, and families, in order to ensure children’s harmonious and adjusted development, thus promoting the quality of life and the well-being of populations.
